# Non-falciparum malaria infections in pregnant women in West Africa

**DOI:** 10.1186/s12936-016-1092-1

**Published:** 2016-01-29

**Authors:** John Williams, Fanta Njie, Matthew Cairns, Kalifa Bojang, Sheick Oumar Coulibaly, Kassoum Kayentao, Ismaela Abubakar, Francis Akor, Khalifa Mohammed, Richard Bationo, Edgar Dabira, Alamissa Soulama, Moussa Djimdé, Etienne Guirou, Timothy Awine, Stephen L. Quaye, Jaume Ordi, Ogobara Doumbo, Abraham Hodgson, Abraham Oduro, Pascal Magnussen, Feiko O. ter Kuile, Arouna Woukeu, Paul Milligan, Harry Tagbor, Brian Greenwood, Daniel Chandramohan

**Affiliations:** Navrongo Health Research Centre, Navrongo, Ghana; Medical Research Council Unit, Fajara, Gambia; London School of Hygiene and Tropical Medicine, Keppel St., London, WC1E 7HT UK; Faculty of Health Sciences, University of Ouagadougou, Ouagadougou, Burkina Faso; Malaria Research and Training Centre, Faculty of Medicine and Odonto-Stomatology, University of Sciences, Techniques and Technologies, Bamako, Mali; JSGlobal, Barcelona Centre for International Health Research (CRESIB), Department of Pathology, Hospital Clinic-Universitat de Barcelona, Barcelona, Spain; Institute of International Health, Immunology and Microbiology, Centre for Medical Parasitology and Institute of Veterinary Disease Biology, University of Copenhagen, Copenhagen, Denmark; Liverpool School of Tropical Medicine, Liverpool, UK; School of Public Health, Kwame Nkrumah University of Science and Technology, Kumasi, Ghana

**Keywords:** Non falciparum malaria, Pregnancy, Africa

## Abstract

**Background:**

Non-*Plasmodium falciparum* malaria infections are found in many parts of sub-Saharan Africa but little is known about their importance in pregnancy.

**Methods:**

Blood samples were collected at first antenatal clinic attendance from 2526 women enrolled in a trial of intermittent screening and treatment of malaria in pregnancy (ISTp) versus intermittent preventive treatment (IPTp) conducted in Burkina Faso, The Gambia, Ghana and Mali. DNA was extracted from blood spots and tested for *P. falciparum*, *Plasmodium vivax*, *Plasmodium malariae* and *Plasmodium ovale* using a nested PCR test. Risk factors for a non-falciparum malaria infection were investigated and the influence of these infections on the outcome of pregnancy was determined.

**Results:**

*P. falciparum* infection was detected frequently (overall prevalence by PCR: 38.8 %, [95 % CI 37.0, 40.8]), with a prevalence ranging from 10.8 % in The Gambia to 56.1 % in Ghana. Non-falciparum malaria infections were found only rarely (overall prevalence 1.39 % [95 % CI 1.00, 1.92]), ranging from 0.17 % in the Gambia to 3.81 % in Mali. Ten non-falciparum mono-infections and 25 mixed falciparum and non-falciparum infections were found. *P. malariae* was the most frequent non-falciparum infection identified; *P. vivax* was detected only in Mali. Only four of the non-falciparum mono-infections were detected by microscopy or rapid diagnostic test. Recruitment during the late rainy season and low socio-economic status were associated with an increased risk of non-falciparum malaria as well as falciparum malaria. The outcome of pregnancy did not differ between women with a non-falciparum malaria infection and those who were not infected with malaria at first ANC attendance.

**Conclusions:**

Non-falciparum infections were infrequent in the populations studied, rarely detected when present as a mono-infection and unlikely to have had an important impact on the outcome of pregnancy in the communities studied due to the small number of women infected with non-falciparum parasites.

## Background

*Plasmodium falciparum* is the dominant malaria parasite in most parts of sub-Saharan Africa and hence responsible for most infections in pregnancy [1]. *Plasmodium vivax* is also a significant cause of maternal morbidity during pregnancy and of low birth weight in parts of Asia [2–5] and Latin America [6, 7], even though the parasite does not sequester in the placenta [8]. *P. vivax* has been reported from time to time in non-pregnant populations in sub-Saharan Africa, especially when polymerase chain reaction (PCR) assays have been used [9–11], but *P. vivax* infections are generally considered to be uncommon in sub-Saharan Africa because of the presence in a high proportion of the population of Duffy antigen negative red cells which cannot be invaded by this malaria parasite. *P. vivax* infections have been recorded only rarely during pregnancy in East or West Africa [12], although they are relatively prevalent in parts of Ethiopia [13]. In contrast, *Plasmodium ovale* and *Plasmodium malariae* are relatively common in many parts of East and West Africa causing a significant proportion of febrile malaria episodes [14] and asymptomatic infections [15]. However, very little is known about the potential impact of non-falciparum malaria infections on pregnancy and there have been very few reports of non-falciparum infections in pregnant women from East or West Africa. Walker-Abbey et al. [16] detected *P. malariae* and *P. ovale* in 6.9 and 1.8 % of pregnant Cameroonian women respectively, nearly always in mixed infections with *P. falciparum*. Mockenhaupt et al. [17] reported an increased susceptibility of pregnant Ghanaian women with alpha + thalassaemia to infection with *P. malariae* and Coldren et al. [18] describe a relapsing case of *P. ovale* in a pregnant women which was probably acquired many years previously in East or West Africa.

Screening of pregnant women at ante-natal clinic (ANC) visits with a rapid diagnostic test (RDT) for malaria and treatment of those who are positive with artemisinin-based combination therapy, an approach termed intermittent screening and treatment (ISTp), is being considered as a potential approach to the control of malaria in some epidemiological situations such as low transmission areas where few women are likely to benefit from currently recommended intermittent preventive treatment with sulfadoxine-pyrimethamine (IPTp-SP). However, there is concern that this approach may lead to some malaria infections, especially those present at low density, being missed and adversely affecting the outcome of pregnancy. This is especially likely to be the case for non-falciparum infections which are often present at only a low density and for which many RDTs have poor sensitivity. For this reason, the prevalence of falciparum and non-falciparum infections at first ANC attendance was investigated in women enrolled in a trial of ISTp versus IPTp-SP conducted in four West African countries (Burkina Faso, The Gambia, Ghana and Mali) and determined how many of these infections were detected by an RDT.

## Methods

Details of the way in which the trial of ISTp versus IPTp-SP was conducted have been presented previously [19]. In brief, 5354 primigravid or secundigravid women were randomised individually at their first ANC visit to the ISTp or IPTp-SP group. Women in the ISTp group were screened for malaria with a RDT at enrollment and at each subsequent routine ANC visit and treated with artemether–lumefantrine (AL) if positive, whilst women in the IPTp group received a dose of SP at each routine ANC visit. Haemoglobin (Hb) concentration was determined at each woman’s final ANC visit, scheduled to occur between 36 and 40 weeks of gestation. Study women were encouraged to deliver in hospital where placental biopsies were obtained for histology and examined using a standardized protocol as described previously [19]. Birth weight was recorded at delivery in women who delivered in hospital and within 7 days of delivery in women who delivered at home. Infants and mothers were evaluated 6 weeks after birth.

The analysis reported in this paper is based on blood samples obtained at the first ANC visit from the 2526 women in the ISTp-AL group. An RDT (First Response^®^ Malaria Rapid Diagnostic Combo Test) was done and blood films and filter paper blots prepared. Blood films were examined for malaria parasites according to the methods described by Swysen et al. [20]. PCR testing was conducted at the MRC Unit, The Gambia. Following extraction of DNA from a filter paper blood spot, the first stage of this nested PCR test involves a PCR assay with the primers rPLU5 + rPLU6. This assay includes positive controls for each of the four main malaria species (*P*. *falciparum*, *P. vivax*, *P. ovale* and *P. malariae*) and a negative control without DNA. The second PCR reaction detects the presence of *Plasmodium* infection using the genus specific primers Plasmo-1 and Plasmo-2. The third stage, undertaken on genus specific samples, determines species by running four parallel PCR assays using the following primers (*P. falciparum*: rFAL 1 + rFAL 2; *P. malariae*: rMAL 1 + rMAL 2; *P. ovale*: rOVA 1 + rOVA 2; *P. vivax*:rVIV 1 + rVIV 2). For further differentiation of *P. ovale* into the two distinct species *P. ovale curtisi* and *P*. *ovale wallikeri*, the *P. ovale* sp. Tryptophan-rich antigen (PoTRA) nested PCR assay was used [21]. Quality control of the assays undertaken in The Gambia was undertaken at Duke University, North Carolina. The lower level of detection of the assay is approximately 10 parasites per µl. The sensitivity of microscopy and an RDT in detecting non-falciparum infections detected by PCR was explored.

The association between demographic and obstetric factors and the presence of falciparum and non-falciparum malaria infections was explored. A multi-variable logistic regression model was then built to explore risk factors for non-falciparum infection, controlling for potential confounding from other risk factors, using those without a malaria infection as the reference group. Women with falciparum malaria infection were excluded from the analysis, as many of the risk factors for *P. falciparum* and non-falciparum infections are likely to be shared. An exploratory analysis was conducted to investigate whether there was any evidence of an association between non-falciparum malaria infections and the primary outcomes of the main trial: active malaria infection of the placenta, low birth weight and haemoglobin at the last ANC contact prior to delivery, again using those with no malaria infection as the reference group. The relationship with measured birth weight, an important secondary end-point for the trial, was also explored.

The trial which provided the samples for this study was approved by the London School of Hygiene and Tropical Medicine Ethical Committee and by the ethics committees of all the partner African institutions: Comité National d’Ethique pour la Recherche en Santé (CNERS), Burkina Faso; Ethics committee of the Ghana Health Service Committee Research & Development Division, Ghana Health Science, Ghana; Navrongo Health Research Centre Institutional Review Board, Ghana; Comité d’éthique de la Faculté de la Faculté de Médecine et d’Odontostomatologie, Mali; The Gambia Government/MRC Laboratories Joint Ethics Committee, The Gambia. Written informed consent was obtained from all participating women. The trial is registered with ClinicalTrials.gov (NCT01084213) and the Pan African Clinical trials Registry (PACT201202000272122).

## Results

PCR detected *P. falciparum* malaria in 981/2526 (38.8 %; 95 % CI 37.0, 40.8) pregnant women at first ANC attendance (Table 1), but only 35 non-falciparum infections were detected, an overall prevalence of 1.39 % (95 % CI 1.00, 1.92) (Table 1). Ten of the non-falciparum infections were mono-infections and 25 were mixed infections with *P. falciparum.* Only three of the 10 mono-infections were detected by RDT and only three by microscopy (two infections were detected by both methods, and one each was detected by RDT and microscopy alone). In two of the latter cases, the microscopic diagnosis was *P. falciparum*. Twenty-three (92 %) of the 25 mixed *P. falciparum* and non-falciparum infections were detected by RDT, and 18 (72 %) were detected by microscopy, with the microscopic diagnosis being *P. falciparum* in all cases. Overall, only nine non-falciparum infections were missed by RDT and 14 missed by microscopy in 2526 pregnant women.

**Table 1:** Falciparum and non-falciparum malaria infections detected in 2526 pregnant West African women at first ANC clinic attendance

	Burkina Faso	Gambia	Ghana	Mali	Total
Number of women	695	604	624	603	2526
Number of *P. falciparum* infections	377	65	350	189	981
Prevalence of *P. falciparum*	54.2 %	10.8 %	56.1 %	31.3 %	38.8 %
(95 % Confidence interval)	(50.5, 57.9)	(8.53, 13.5)	(52.2, 59.9)	(27.8, 35.2)	(37.0, 40.8)
Number of non-falciparum malaria infections overall	5	1	6	23	35
Prevalence of non-falciparum malaria	0.72 %	0.17 %	0.96 %	3.81 %	1.39 %
(95 % Confidence interval)	(0.30, 1.72)	(0.02, 1.17)	(0.43, 2.13)	(2.55, 5.68)	(1.00, 1.92)
Number of mono-infections					
*P. falciparum*	374	65	345	172	956
*P. malariae*	0	0	0	3	3
*P. vivax*	0	0	0	2	2
*P. ovale curtisi*	1	0	0	1	2
*P. ovale wallikeri*	1	1	1	0	3
Mixed infections					
*P. falciparum*, *P. malariae*	0	0	1	10	11
*P. falciparum*, *P. ovale curtisi*	1	0	1	1	3
*P. falciparum*, *P. ovale wallikeri*	1	0	2	3	6
*P. falciparum*, *P. vivax*	0	0	0	2	2
*P. falciparum*, *P. malariae*, *P. ovale curtisi*	0	0	1	1	2
*P. falciparum*, *P. malariae*, *P. ovale wallikeri*	1	0	0	0	1

*Plasmodium malariae* was the most common non-falciparum species detected, being present alone in three women (all from Mali) and in 14 mixed infections (11 with *P. falciparum*, and three with *P. falciparum* and *P. ovale*). Eleven mixed *P. ovale* and *P. falciparum* infections were detected. Both forms of *P*. *ovale* were found in roughly equal abundance (seven *P. ovale curtisi*, nine *P. ovale wallikeri*, and one either mixed infection of *P. ovale curtisi* with *P. ovale wallikeri*, or an indeterminate *P. ovale* infection). *P.vivax* was found in only four women (two mixed with *P. falciparum*), all of whom lived in Mali. Because of the unexpected finding of *P. vivax* parasites in four black, African women resident near Bamako, the identity of these parasites was confirmed using a real-time PCR assay targeting the *P. vivax* ribosomal RNA gene (S.R. Meshnick, personal communication).

There was variation in the prevalence of non-falciparum malaria between study sites, with Mali having the highest prevalence (3.81 % [95 % CI 2.55, 5.68]) compared with Ghana (0.96 % [95 % CI 0.43, 2.13]), Burkina Faso (0.72 % [95 % CI 0.30, 1.72]) and The Gambia (0.17 % [95 % CI 0.02, 1.17]).

There were no major differences in the prevalence of non-falciparum malaria infections overall according to gravidity, gestational age at enrolment, educational level or socio-economic status (Table 2). There was some suggestion of differences in the prevalence of non-falciparum malaria between age groups, although the statistical evidence for this was not strong. Relative to mothers below 18 years of age, the adjusted odds ratio [OR] for mothers aged 18–20 years was 3.77 (95 % CI 1.03, 13.8) (*p* = 0.045) but there were no differences in older age groups compared to mothers below 18 years of age. Non-falciparum malaria infections were more common among women in the two poorest socio-economic groups; adjusted OR relative to the wealthiest group (6.48 [95 % CI 1.68, 25.0] and 6.55 [95 % CI 1.27, 33.7]) in the poor and poorest groups respectively. As found in the previous analysis of *P. falciparum* infections in study women [16], non-falciparum malaria infections were more common among women who were enrolled in the late wet season (between September and November): adjusted OR 7.10 (95 % CI 2.56, 19.7), *p* < 0.001 (Table 3). Following adjustment for these other factors, the association between non-falciparum malaria and study centre was strengthened: relative to Ghana (the centre with the second highest prevalence), the adjusted OR for Mali was 6.54 (95 % CI 1.79, 23.9), *p* = 0.005 and for The Gambia 0.10 (95 % CI 0.01, 0.88) (*p* = 0.038). There remained no differences between Ghana and Burkina Faso, adjusted OR 0.53 (95 % CI 0.14, 2.03) (*p* = 0.353).

**Table 2:** The prevalence of *P. falciparum* and non-falciparum malaria infections overall at first ANC visit according to baseline characteristics

	Uninfected		*P. falciparum infection*		Non-falciparum infection	
	No.	%	No.	%	No.	%
Gravidity						
Primigravid	754	54.7	605	43.9	20	1.5
Secundigravid	736	64.8	385	33.9	15	1.3
Gestational age^a^						
<20 weeks	510	55.6	397	43.3	10	1.1
20–24 weeks	794	59.9	509	38.4	23	1.7
25–30 weeks	187	68.0	86	31.3	2	0.7
Age						
Under 18 years	194	61.4	119	37.7	3	0.9
18–20 years	656	53.8	542	44.4	22	1.8
21–24 years	434	64.4	234	34.7	6	0.9
25 years +	211	66.8	101	32.0	4	1.3
Educational level^b^						
1-none	689	59.6	452	39.1	15	1.3
2-basic	557	56.3	416	42.0	17	1.7
3-secondary	210	65.8	107	33.5	2	0.6
4-tertiary	20	57.1	14	40.0	1	2.9
Season enrolled^c^						
Early wet (6–8)	614	69.6	261	29.6	7	0.8
Late wet (9–11)	188	45.4	215	51.9	11	2.7
Early dry (12–2)	251	46.5	282	52.2	7	1.3
Late dry (3–5)	442	64.1	238	34.5	10	1.4
Socio-economic status^d^						
1-wealthiest	375	77.8	99	20.5	8	1.7
2-wealthy	325	67.8	142	29.6	12	2.5
3-medium	262	54.5	217	45.1	2	0.4
4-poor	230	48.0	241	50.3	8	1.7
5-poorest	226	46.1	259	52.9	5	1.0

^a^Gestational age at enrolment assessed by symphysis-fundal height

^b^Educational level reported by the mother at the enrolment visit

^c^Season enrolled indicates the month of the year when the woman first presented to ANC and was enrolled into the study

^d^Socio-economic status defined using principal components analysis of durable household assets and household amenities, as described in [16]

**Table 3:** Risk factors for non-falciparum malaria infections overall at first ANC attendance

Risk factor	Crude OR (95 % CI)	*p* value	Adjusted OR (95 % CI)	*p* value
Country				
Ghana	[Reference]	–	[Reference]	–
Burkina Faso	0.74 (0.22, 2.46)	0.627	0.53 (0.14, 2.03)	0.353
Gambia	0.08 (0.01, 0.69)	0.021	0.10 (0.01, 0.88)	0.038
Mali	2.52 (1.01, 6.28)	0.047	6.54 (1.79, 23.9)	0.005
Gravidity				
Primigravid	[Reference]	–	[Reference]	–
Secundigravid	0.85 (0.42, 1.69)	0.639	0.71 (0.33, 1.54)	0.386
Gestational age				
<20 weeks	[Reference]	–	[Reference]	–
20–24 weeks	1.51 (0.70, 3.23)	0.290	1.92 (0.85, 4.31)	0.115
25–30 weeks	0.57 (0.12, 2.64)	0.469	0.60 (0.12, 2.90)	0.521
Age				
Under 18 years	[Reference]	–	[Reference]	–
18–20 years	2.98 (0.86, 10.3)	0.084	3.77 (1.03, 13.8)	0.045
21–24 years	1.38 (0.32, 5.83)	0.665	2.14 (0.43, 10.6)	0.351
25 years +	1.75 (0.36, 8.41)	0.484	2.54 (0.45, 14.3)	0.290
Education level				
1-none	[Reference]	–	[Reference]	–
2-basic	0.92 (0.43, 1.96)	0.833	0.96 (0.41, 2.22)	0.924
3-secondary	0.27 (0.06, 1.23)	0.092	0.27 (0.05, 1.32)	0.106
4-tertiary	1.36 (0.17, 11.2)	0.774	3.73 (0.35, 40.2)	0.277
Season enrolled				
Early wet (6–8)	[Reference]	–	[Reference]	–
Late wet(9–11)	5.59 (2.10, 14.9)	0.001	7.10 (2.56, 19.7)	<0.001
Early dry (12–2)	2.84 (0.97, 8.27)	0.056	2.54 (0.84, 7.67)	0.098
Late dry(3–5)	1.76 (0.66, 4.69)	0.260	1.85 (0.68, 5.04)	0.228
SES				
1-wealthiest	[Reference]	–	[Reference]	–
2-wealthy	2.14 (0.85, 5.34)	0.105	1.82 (0.68, 4.83)	0.230
3-medium	1.07 (0.21, 5.33)	0.935	0.96 (0.18, 5.02)	0.963
4-poor	6.30 (1.83, 21.7)	0.004	6.48 (1.68, 25.0)	0.007
5-poorest	5.89 (1.35, 25.7)	0.018	6.55 (1.27, 33.7)	0.024

Crude ORs for gravidity, gestational age, age, education level, season of enrolment, and social economic status are adjusted for country as an a priori covariate. Adjusted ORs presented adjusted for all other covariates in the multivariable model. Gestational age at enrolment was assessed by symphysis-fundal height. Educational level reported by the mother at the enrolment visit. Season enrolled indicates the month of the year when the woman first presented to ANC and was enrolled into the study. Socio-economic status defined using principal components analysis of durable household assets and household amenities, as described in [16]

The prevalence of active placental malaria infection at delivery was similar in those with a mono- or mixed non-falciparum malaria infection and those with no infection: 23.1 versus 21.8 % respectively, adjusted OR 1.63 (95 % CI 0.58, 4.60), *p* = 0.356 (Table 4). Of the six women with a non-falciparum mono infection who also had a placental malaria result, only two had an active infection of the placenta (acute in both cases) and the infection was not noted to be one with a non-falciparum infection. Sections from four of these women showed pigment in fibrin and/or macrophages (three without malaria infection, one with malaria infection) and one mild inflammation in a woman with malaria infection. Prevalence of low birth weight was similar between those with a mono- or mixed non-falciparum malaria infection and those with no malaria infection: 16.1 and 17.1 % respectively, adjusted odds ratio 0.95 (95 % CI 0.35, 2.58) (*p* = 0.915). Similarly, there was no evidence of a difference in mean birth weight between women with a mono- or mixed non-falciparum infection and those with no malaria infection during pregnancy: adjusted difference—51.9 g (95 % CI −220.8 g, +117.0 g) (*p* = 0.547), Table 4. Finally, measured Hb at the final ANC contact was also similar in those with no malaria infection (10.93 g/dL) and in those with a mono- or mixed non-falciparum infection (11.05 g/dL): adjusted difference 0.01 g/dL (95 % CI −0.55, + 0.56) (*p* = 0.983).

**Table 4:** Association of non-falciparum malaria overall with birth outcomes including placental malaria (PM), birth-weight and haemoglobin concentration

	No malaria infection		Non-falciparum infection	
	No.	%	No.	%
No active PM	744	78.2	20	76.9
Active PM	207	21.8	6	23.1
Crude OR (95 % CI), *p* value	[Reference]	–	1.06 (0.41, 2.76)	0.90
Adjusted OR (95 % CI), *p* value	[Reference]	–	1.63 (0.58, 4.60)	0.36
Normal birth weight	1099	82.9	26	83.9
Low birth weight	227	17.1	5	16.1
Crude OR (95 % CI), *p* value	[Reference]	–	0.97 (0.36, 2.57)	0.94
Adjusted OR (95 % CI), *p* value	[Reference]	–	0.95 (0.35, 2.58)	0.92

	Mean	SD	Mean	SD
Birth weight (kg)	2.85	0.48	2.78	0.31
Crude difference, grammes (95 % CI), *p* value	[Reference]	–	−57.7 (−227.7, 112.3)	0.51
Adjusted difference, grammes (95 % CI), *p* value	[Reference]	–	−51.9 (−220.8, 117.0)	0.54
Hb at final ANC	10.93	1.36	11.05	1.16
Crude difference, g/dL (95 % CI), *p* value	[Reference]	–	−0.09 (−0.64, 0.47)	0.76
Adjusted difference, g/dL (95 % CI), *p* value	[Reference]	–	0.01 (−0.55, 0.56)	0.98

Crude odds ratios/differences are adjusted for country as an a priori covariate. Adjusted odds ratios/differences also adjust for gravidity, gestational age, age, education level, season of enrolment, and social economic status

## Discussion

This study confirmed that non-falciparum malaria infections are present in pregnant women in West Africa but that they are uncommon, with an overall prevalence across four countries of 1.4 % at first ANC attendance. It is likely that some additional infections occurred later in pregnancy. However, among the women from Ghana enrolled in this study who were followed throughout pregnancy, at least a half of all malaria infections were detected at the first ANC visit [22]; the figures for all ANC visits were 53.6 % for RDT, 60.6 % for microscopy and 64.3 % for PCR respectively. Thus, unless there is a differential rate of infection between non-falciparum and falciparum infections, it is likely that the prevalence of non-falciparum infections at any time point during the course of pregnancy would have been be low in the study population. Prevalence of non-falciparum infections varied between centres, being less than 1 % in three, but close to 4 % in Mali. These differences cannot be explained solely by differences in the intensity of transmission between centres as Mali had the second lowest prevalence of *P. falciparum* infection of the four study sites. *P. vivax* was detected only in Mali, and only in one sub-site in suburban Bamako. This malaria parasite is known to be present in northern Mali [10], perhaps because of some mixing with north African populations, but not in the southern parts of the country where all these infections were found. This unexpected finding is being followed up further.

The results of this study indicate the difficulty in identifying non-falciparum malaria infections by microscopy when these infections are encountered rarely and often present in mixed infections with *P. falciparum*: only three of the 10 pure non-falciparum infections were detected by microscopy, and two of these were mistaken for *P. falciparum* despite the fact that study slides were read by two well-trained microscopists and that the quality of microscopical reading was well controlled. In sub-Saharan Africa, microscopists expect malaria infections present in a blood film to be due to *P. falciparum* and this may have influenced their recording of the results. Rapid diagnostic tests did not perform any better than microscopy, also missing seven of the 10 non-falciparum mono-infections, perhaps because these were present at only a low density. When *P. falciparum* was also present, RDTs detected all but two of these infections, whereas microscopy missed seven of the mixed infections. These findings suggest that when, in sub-Saharan Africa, detection of non-falciparum malaria infection in pregnancy depends upon microscopy, many non-falciparum infections will be missed. Factors likely to have contributed to the poor performance of microscopy and a RDT in detecting non-falciparum infections are their frequent occurrence as mixed infections with *P. falciparum* and their presence at only a low density. Why the latter should be the case is uncertain, but it may be due in part to suppression of the minor parasite by *P. falciparum* in mixed infections and in part due to the fact these may have been more chronic infections than those caused by *P. falciparum.*

Enrolment at the end of the wet season was a risk factor for non-falciparum malaria infection, as was lower socio-economic status. These are both well-established risk factors for malaria infection in general. The suggestion of an association of one specific age group with higher risk of non-falciparum infections does not have a biological explanation and is likely to be a chance finding as the numbers of non-falciparum infections studied was small.

There was no association between the presence of a non-falciparum malaria infection at first ANC presentation and an adverse outcome of pregnancy. However, the number of non-falciparum infections was small, 26 of the 35 individuals with non-falciparum infections at baseline were treated with artemether-lumefantrine and it is possible that some women in the uninfected reference group were infected at later time points, diluting differences between the two groups, so this finding is not surprising.

This study was undertaken as part of an evaluation of ISTp because of concerns that a significant number of infections caused by non-falciparum species might be missed during RDT testing, with an adverse impact on the outcome of pregnancy, if this approach to malaria control in pregnancy was to be implemented. Because the majority of non-falciparum infections occurred in conjunction with a falciparum infection which was detected by a RDT, as noted in another recent study in Burkina Faso and Uganda (Hopkins et al. personal communication), the majority of women with a non-falciparum infection received appropriate treatment; only seven non-falciparum mono-infections and two mixed non-falciparum and falciparum infections were missed at first ANC visit using a ‘combo’ RDT among 2526 women. For this reason the occurrence of non-falciparum infections is unlikely to be a concern should ISTp be recommended for any population group in sub-Saharan Africa. However, this might not be the case in other parts of the malaria endemic world where non-falciparum infections, particularly those caused by *P. vivax*, are more prevalent and where many more infections might be missed using currently available RDTs.
